# Growth and Clinical Outcomes Before and After Implementation of a Donor Human Milk Program in Very Preterm and/or Very-Low-Birth-Weight Infants

**DOI:** 10.3390/nu18203338

**Published:** 2026-10-12

**Authors:** Jacky Herzlich, Omer Less, Itamar Gilboa, Yaffa Elbaum-Shiff, Ariel Halperin

**Affiliations:** 1 Department of Neonatology, Dana Dwek Children’s Hospital, Tel Aviv Sourasky University Medical Center, Tel Aviv 6423906, Israel; arielha@tlvmc.gov.il; 2 Gray Faculty of Medical and Health Sciences, Tel Aviv University, Tel Aviv 6997801, Israel; omerless123@gmail.com (O.L.); itamar.glb@gmail.com (I.G.); yaffas@tlvmc.gov.il (Y.E.-S.); 3 Department of Obstetrics and Gynecology, Lis Maternity Hospital, Tel Aviv Sourasky University Medical Center, Tel Aviv 6423906, Israel; 4 Department of Dietetics and Nutrition, Tel Aviv Sourasky University Medical Center, Tel Aviv 6423906, Israel

**Keywords:** donor human milk, mother’s own milk, extremely low birth weight, enteral feeding, parenteral nutrition, necrotizing enterocolitis

## Abstract

**Background/Objectives**: Mother’s own milk (MOM) is widely recognized as the best source of nutrition for premature infants, with donor human milk (DHM) as the next recommended option. However, it remains controversial whether DHM confers the same benefits as MOM. We aimed to compare growth and clinical outcomes in very-low-birth-weight (VLBW) infants before and after the introduction of DHM in our Neonatal Intensive Care Unit (NICU). **Methods**: This is a retrospective cohort of VLBW and/or very-low-birth-weight infants born between January 2018 and June 2023. Infants were divided based on birth date into Epoch-1 (pre-DHM, N = 98) and Epoch-2 (post-DHM, N = 138). Feeding groups included exclusive MOM (N = 112), MOM + DHM (N = 71), and MOM + Formula (N = 39). Baseline characteristics were compared between groups. The primary outcome was weight gain; secondary outcomes included incidence of necrotizing enterocolitis (NEC), time to full enteral feeding, and length of hospitalization. Multivariable regression models were used to assess these outcomes while adjusting for relevant confounders. **Results**: Infants in Epoch-2 were born at a significantly higher gestational age (29.3 vs. 28.2 weeks, *p* < 0.001) and had a higher median birth weight (1350 g vs. 1080 g, *p* < 0.001) compared to Epoch-1. In this before-and-after cohort, the post-DHM implementation epoch was not independently associated with differences in growth or complication rates after adjustment. Time to reach full enteral feeding was significantly shorter in the post-DHM epoch (B = −0.255, *p* < 0.001). **Conclusions**: DHM had no independent effect on growth or complication rates but was associated with earlier achievement of full enteral feeding. Further studies are needed to evaluate its contribution to neonatal clinical outcomes.

## 1. Introduction

Premature birth, occurring before 37 weeks of gestation, is a leading cause of neonatal and infant morbidity and mortality [1,2]. Preterm infants are at a significant risk of complications, with serious long-term consequences, particularly for those born very preterm or VLBW [1]. Compared to full-term newborns, preterm infants have fewer energy reserves at birth and are more vulnerable to stressors such as respiratory distress or infection, which further increase metabolic demands [3]. Early nutritional support, aimed at meeting the increased nutritional requirements of preterm infants and supporting appropriate postnatal growth and nutrient accretion, is a critical determinant of outcomes [1,3,4]. Expert and guideline groups consistently recommend MOM as the primary source of nutrition for premature infants [1,5,6]. DHM is an additional feeding option when there is insufficient breast milk or the mother is unable to breastfeed [1,5]. These recommendations are based on the well-established benefits of human milk (HM) [7,8,9], including protection against infection and NEC [10,11,12], a severe gastrointestinal disease, primarily affecting preterm infants [10,13], and associated with longer hospitalization [14]. These protective effects appear dose-dependent and are attributed collectively to the interaction of multiple bioactive components rather than any single nutrient [11]. HM has also been associated with shorter NICU stays, development of a healthy neonatal gut microbiome, and favorable brain, visual, and cognitive development. These associations are likely related to the combined effects of multiple nutritional and bioactive components of human milk, including essential fatty acids and their precursors to long-chain polyunsaturated fatty acids (LC-PUFAs) [15]. DHM serves as an alternative when MOM is unavailable, but given its limited availability, DHM is typically reserved for the smallest and sickest infants [16]. To facilitate the process between donors and recipients, human milk banks are expanding worldwide [17,18]. However, clinical evidence remains limited [17], and practices and regulatory frameworks vary substantially [16,18].

Here, we report our experience from a large medical center NICU following the establishment of a human milk bank and the introduction of DHM into the feeding protocol in August 2020. We aimed to evaluate growth and gastrointestinal outcomes among VLBW and very preterm infants before and after the introduction of DHM as part of the feeding regimen.

## 2. Materials and Methods

### 2.1. Study Design and Participants

This retrospective study represents a secondary analysis of a previously described VLBW and/or very-low-birth-weight cohort (<1500 g birth weight or gestational age < 32 weeks) [19] managed according to the unit’s DHM policy. The study included preterm infants admitted to our NICU between 1 January 2018 and 30 June 2023, with birth weight (BW) below 1500 g or at gestational age (GA) below 32 weeks. Infants were classified into two epochs based on the timing of DHM introduction into the feeding protocol. DHM in the national bank undergoes Holder pasteurization and freezing. Epoch-1 preceded DHM availability (prior to August 2020), during which infants received MOM, formula, or MOM supplemented with formula. Epoch-2 followed DHM introduction (from August 2020 onward), during which infants received MOM or MOM supplemented with DHM. The criteria for enteral feeding advancement, fortification, and withholding of feeds remained unchanged during the study period. Nevertheless, the availability of DHM provided clinicians with an additional human milk option. In clinical practice, attending physicians reported greater comfort with initiating and advancing enteral feeding when MOM was unavailable because DHM was available as an alternative to formula. Infants were excluded if they had chromosomal abnormalities, died during hospitalization, or received feeding regimens outside the defined protocol. The study was performed in line with the principles of the Declaration of Helsinki. Approval was granted by the “Helsinki Committee” (0172-22-TLV, 8 April 2022).

### 2.2. Data Collection

Maternal and neonatal data were extracted from medical records, including characteristics, complications, and interventions. NEC was defined as Bell’s stage II or higher, and feeding intolerance was defined as gastric residual volume ≥20% of the preceding feed. Other clinical findings, including abdominal distension, emesis, or bloody stools, were not required for classification because they were not consistently recorded as components of this variable, and full enteral feeding was defined as enteral intake ≥140 mL/kg/day. Intraventricular hemorrhage (IVH) was graded according to the Papile classification and defined as present if grade ≥ 1. Retinopathy of prematurity (ROP) was classified according to the ICROP criteria. Small for gestational age (SGA) status was classified according to Dollberg’s chart. Significant respiratory support included mechanical ventilation and/or nasal cannula support.

### 2.3. Study Outcomes

The primary outcome was weight gain over the first 28 days as the primary outcome, assessed at predefined time points, expressed as the percentage increase from birth weight (BW). Secondary outcomes included the incidence of NEC, time to reach full enteral feeding, and length of hospitalization across the two epochs.

### 2.4. Statistical Analysis

Categorical variables were summarized as frequencies and percentages. Continuous variables were assessed for normality using the Shapiro–Wilk test and reported as means ± standard deviations or medians with interquartile ranges, as appropriate. Comparisons of continuous variables between groups were performed using Mann–Whitney U, Kruskal–Wallis, or one-way ANOVA tests. Post hoc analyses were conducted with Bonferroni correction for multiple comparisons, and adjusted *p*-values are reported, as appropriate. Categorical variables were compared using Chi-square or Fisher’s exact tests. Boxplots were used to visually represent the distribution of continuous variables. Because day 14 was the only timepoint at which a significant epoch difference was observed, a multiple linear regression model was fitted for weight gain at this timepoint. The model was adjusted for GA, BW, gender, feeding intolerance, epoch, and timing of enteral feeding initiation. Additionally, multiple linear regression analyses were performed to evaluate time to achieve full enteral feeding and length of hospital stay. The model for time to achieve full enteral feeding was adjusted for GA, BW, gender, feeding intolerance, NEC, epoch, and timing of enteral feeding initiation. The model for length of hospital stay was adjusted for GA, BW, NEC, BPD, and epoch. A logistic regression model was used for the categorical outcome of NEC (Supplemental Tables S1–S4) The regression models were conducted as exploratory analyses to identify associations, as the retrospective design precludes establishing temporality. All analyses included the complete final study cohort. All analyses were performed using IBM SPSS Statistics for Windows, version 29. A *p*-value < 0.05 was considered statistically significant.

## 3. Results

### 3.1. Study Population

**Figure 1:**
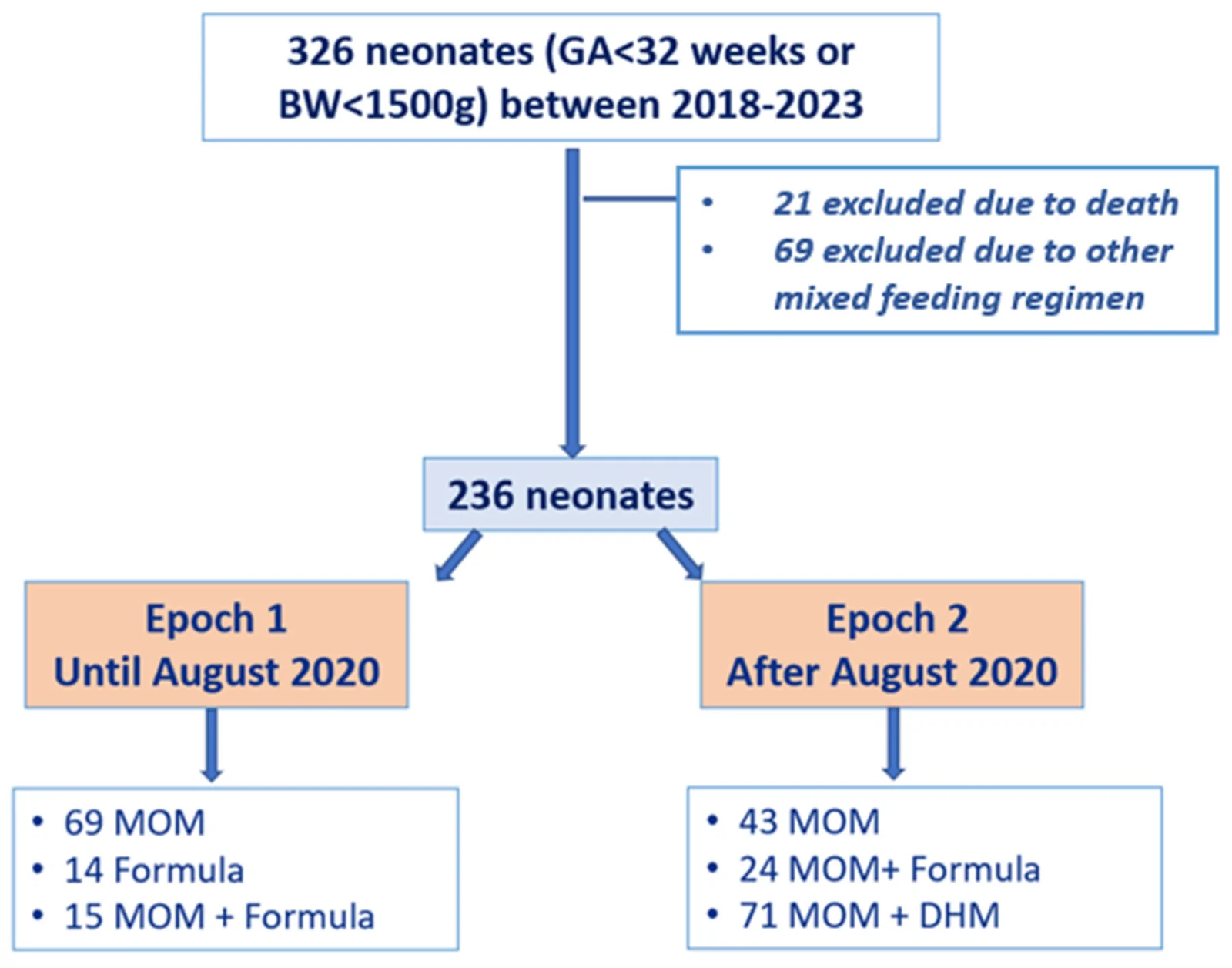
Flowchart of study cohort.

Of 326 neonates born between 2018 and 2023, 236 met all inclusion criteria and were included in the final analysis: 98 in Epoch-1 and 138 in Epoch-2, 69 babies were excluded because they did not receive MOM, MOM+DHM, or MOM+Formula (Figure 1). In Epoch-2, they had a mixed regimen including formula with DHM and MOM all 3 together. The 21 who died were excluded as stated in the exclusion criteria; we did not fear survivor bias because 10 were in Epoch-1 and 11 were in Epoch-2.

### 3.2. Demographic and Feeding Characteristics

Comparison of baseline characteristics revealed that infants in Epoch-2 were born at a significantly higher GA (29.3 vs. 28.2 weeks, *p* < 0.001) and had a higher median BW (1350 g vs. 1080 g, *p* < 0.001) compared to Epoch-1. Mode of delivery also differed significantly between epochs (*p* = 0.010), with higher vaginal delivery rates in Epoch-2. Antenatal corticosteroid use was significantly higher in Epoch-2 (80.4% vs. 65.3%, *p* = 0.009). In Epoch-1, the majority, 69 infants (70.4%), received exclusive MOM, while 14 (14.3%) received exclusive formula and 15 (15.3%) received a combination of MOM and formula. In Epoch-2, the majority, 71 infants (51.4%), received a combination of MOM and DHM, while 43 (31.2%) received exclusive MOM, and 24 (17.4%) received a combination of MOM and formula. Maternal demographics and neonatal characteristics are summarized in Table 1.

### 3.3. Clinical Outcomes and Growth-Related Parameters

Rates of significant respiratory support (78.9% vs. 96.9%), surfactant administration (35.5% vs. 58.1%), and RDS (75.3% vs. 93.8%) were significantly lower in Epoch-2 compared to Epoch-1 (*p* < 0.001 for all). Similarly, BPD (41.3% vs. 56.1%, *p* = 0.025) and feeding intolerance (22.4% vs. 34.6%, *p* = 0.038) were significantly lower in Epoch-2. No significant differences were observed between epochs in the rates of NEC, SIP, BSI, PVL, IVH, hypoglycemia, or ROP.

Enteral feeding was initiated significantly earlier in Epoch-2 (1.5 vs. 2.3 days, *p* < 0.001). Percentage weight change at day 14 was significantly higher in Epoch-2 (9% vs. 4.9%, *p* = 0.019), while percentage weight change at days 7 and 28 did not differ significantly between epochs. Infants in Epoch-2 achieved full enteral feeding earlier (12.8 vs. 26.8 days, *p* < 0.001), required shorter TPN duration (9 vs. 20 days, *p* < 0.001), and had shorter length of hospitalization (58.7 vs. 72.8 days, *p* < 0.001). Clinical outcomes and growth-related parameters are presented in Table 2.

### 3.4. Multivariable Analysis of Growth and Clinical Outcomes

#### 3.4.1. Weight Gain at Day 14

At day 14, GA (B = 0.566, *p* < 0.001), female gender (B = 0.123, *p* = 0.042), and earlier initiation of enteral feeding (B = 0.144, *p* = 0.035) were significantly associated with weight gain, while BW (B = −0.379, *p* < 0.001) was negatively associated. Notably, no significant epoch effect was observed for weight gain at day 14. The model explained 16.0% of the variance in weight change at day 14 (Adjusted R^2^ = 0.160).

#### 3.4.2. Length of Hospitalization

NEC (B = 0.114, *p* = 0.006) and BPD (B = 0.147, *p* = 0.008) were significantly associated with hospitalization, whereas GA (B = −0.521, *p* < 0.001) and BW (B = −0.211, *p* < 0.001) were negatively associated. No significant epoch effect was observed for length of hospitalization. The model explained 64.5% of the variance in length of hospitalization (Adjusted R^2^ = 0.645).

#### 3.4.3. Time to Reach Full Enteral Feeding

Epoch was significantly and negatively associated with time to full enteral feeding (B = −0.255, *p* < 0.001), indicating earlier time to full enteral feeding in Epoch-2 compared to Epoch-1. Feeding intolerance (B = 0.155, *p* = 0.006) and NEC (B = 0.230, *p* < 0.001) were significantly associated with time to full enteral feeding, indicating a prolonged time to full enteral feeding. The model explained 33.5% of the variance in time to reach full enteral feeding (Adjusted R^2^ = 0.335).

### 3.5. Incidence of NEC

Duration of TPN (OR = 1.123, *p* < 0.001), feeding intolerance (OR = 3.993, *p* = 0.010), and GA (OR = 1.390, *p* = 0.038) were significantly positively associated with NEC, whereas epoch, gender, SGA status, and initiation of enteral feeding were not significantly associated. The model explained 51.7% of the variance in NEC occurrence (Nagelkerke R^2^ = 0.517).

## 4. Discussion

This study evaluated growth and gastrointestinal outcomes in VLBW and very preterm infants, before and after the introduction of DHM supplementation during their stay in the NICU. Overall, weight gain did not differ significantly between epochs; an initial difference observed at day 14 was no longer significant after adjustment. Length of hospitalization and NEC incidence were also comparable between epochs. However, time to reach full enteral feeding was significantly shorter in the post-DHM epoch.

Growth patterns of preterm infants are not entirely understood [20]. Early weight fluctuations are typical, primarily resulting from changes in fluid balance [21]. Once nutritional tolerance is established, extremely preterm infants often show accelerated growth and generally achieve catch-up growth by 3 years corrected age [20,21]. The literature suggests that DHM is associated with lower short-term weight gain compared with formula [8,22,23,24]. In our cohort, however, weight gain did not differ significantly before and after DHM introduction. Although differences in study design, outcome measures, and time point limit direct comparison, this is broadly consistent with a recent multicenter randomized trial in extremely preterm infants, which found no evidence of inferiority of DHM compared with formula with respect to growth at 2 years corrected age [25]. Regardless of feeding source, preterm infant growth is strongly dependent on adequate protein intake [26,27]. Protein concentration is naturally high in preterm milk but, since preterm MOM is produced in lower volumes, the protein supply might be insufficient and often requires supplementation [11]. Protein content in DHM is inherently heterogeneous, reflecting differences among donors in gestational age, lactation stage, and maternal diet [17,28]. Unlike MOM, which adapts dynamically to the infant’s evolving needs, DHM is often sourced from term donors at later stages of lactation, when protein content is naturally lower [17,29]. DHM processing further affects composition and bioactivity [8]. Processing stages include pasteurization to inactivate bacteria and viruses, fat homogenization to disperse lipid droplets, and freezing and thawing [30]. For example, fat absorption is reduced due to loss of lipase activity and changes in fat globule structure [8]. DHM heterogeneity may also compromise the protective role of HM against NEC. In general, HM is associated with a lower risk of NEC compared to formula, an effect attributed to the presence of protective components in MOM rather than harmful components in formula [31]. However, it remains controversial whether DHM confers the same protection [10]. Immune cells and other bioactive components in MOM likely contribute to protection against NEC and other morbidities. As these components are inactivated during DHM processing and largely absent from formula, their protective effect may be reduced or lost [29]. Additionally, unlike direct breastfeeding, DHM, formula, and expressed MOM lack the bidirectional suckling feedback loop, which may further limit immune adaptation and reduce immune cell activity [29]. Fatty acids represent another important nutritional element. Essential fatty acids, obtained solely from the diet, contribute to immune system function, alongside their role in neurological development. However, LC-PUFA concentrations in MOM decline over the course of lactation. Pasteurization appears to preserve fatty acid profiles, but freezing may reduce fat content. As with protein, these donor- and processing-related factors may affect fatty acid concentrations in DHM. Formula manufacturers have attempted to replicate MOM fatty acid profile using vegetable fat blends, but structural differences persist, and optimizing formula fat composition remains an active area of research [29]. While previous studies have found lower rates of NEC with DHM, albeit with slower growth [22,30,32,33], NEC rates did not differ significantly between epochs in our cohort, possibly reflecting a reduced protective effect related to processing and donor factors. We also found feeding intolerance and TPN duration positively associated with NEC. This is consistent with the literature suggesting an association between prolonged reliance on parenteral nutrition, limited enteral stimulation, and NEC risk, potentially involving mucosal atrophy and dysbiosis [34,35]. Unexpectedly, GA was also positively associated with NEC, contrary to the well-established inverse relationship typically reported in the literature [13]. This counterintuitive finding remains unexplained and should therefore be interpreted with caution. It may reflect specific cohort characteristics and warrants further investigation. NEC and BPD were positively associated with hospitalization, while GA and birth weight were negatively associated. Evidence suggests a bidirectional relationship in which neonatal morbidities impair adequate nutritional delivery, resulting in suboptimal growth, which may in turn delay recovery and prolong hospitalization [21]. Feeding intolerance was positively associated with time to full enteral feeding, consistent with evidence that faster advancement of enteral feeding reduces time to full enteral feeding [6]. Moreover, infants in the post-DHM epoch also achieved full enteral feeding significantly earlier, potentially implying a mechanism whereby DHM improves feeding tolerance, leading to a shorter time to full enteral feeding. Although we did not directly test whether DHM was an independent predictor of feeding intolerance in our cohort, this mechanism is supported by previous evidence showing that DHM reduces feeding intolerance [30,36]. Future randomized trials are needed to establish causality and investigate potential mediating factors.

These findings contribute to the ongoing debate regarding DHM implementation in clinical practice. This is further illustrated by a recent national survey of U.S. NICUs, which found that although DHM is used by most facilities, substantial variation in feeding practices persists, with no consensus on optimal strategies for extremely preterm infants [37].

### Strengths and Limitations

Strengths of this study include a well-defined cohort of VLBW or very preterm infants and the clear division into two study epochs, based on a distinct clinical policy change (introduction of milk bank). The inclusion of both unadjusted comparisons and multivariate regression models enhances the robustness of the findings and helps isolate the contribution of individual predictors. However, several limitations should be acknowledged. As a retrospective study, incomplete or inconsistent documentation may have introduced information bias. The selection of day 14 for multivariable analysis was data-driven, as this was the only timepoint showing a significant epoch difference in the univariate analysis, and should therefore be interpreted with caution. A longitudinal mixed-effects model incorporating all timepoints simultaneously may have provided a more appropriate approach. Selection bias is a potential concern, as infants were not randomly allocated to groups. Epoch-1 included infants with lower BW, potentially indicating a higher baseline risk for complications. Despite this, no significant difference in NEC incidence was observed between the two study epochs. Given the observational design and differences in feeding exposure and baseline characteristics, these findings should not be interpreted as evidence of equivalence between formula and DHM. Although the sample included over 200 infants, the power to detect small differences in rare outcomes such as NEC remained limited [22]. Larger, prospective studies may be required to evaluate the effect of DHM more definitively on these complications. Residual confounding: Despite the use of multivariate models, unmeasured factors such as staff experience, parental involvement, or nutritional practices (e.g., use of fortifiers) may have influenced outcomes.

## 5. Conclusions

The introduction of donor human milk in our NICU did not independently affect growth or complication rates. Observed differences between epochs were mainly related to infant maturity and clinical severity rather than DHM itself. DHM appears to be an alternative to formula, but further studies are needed to evaluate its long-term effects and to define its specific contribution to neonatal outcomes compared to formula.

## Figures and Tables

**Table 1:** Neonatal and maternal characteristics.

Variables	Epoch-1 (n = 98)	Epoch-2 (n = 138)	*p*-Value
GA, weeks	28.2 (±1.8)	29.3 (±2.5)	<0.001
**Delivery mode**			0.010
Vaginal delivery	24 (24.4)	54 (39.1)	
Emergency CS	74 (75.5)	80 (57.9)	
Elective CS	0 (0)	4 (2.8)	
Twins	24 (24.4)	43 (31.1)	0.263
Gender Female	55 (56.1)	79 (57.2)	0.864
IUGR	19 (19.3)	17 (12.3)	0.143
IDM	6 (6.1)	19 (13.7)	0.060
Antenatal corticosteroid treatment	64 (65.3)	111 (80.4)	0.009
Pre-eclampsia	15 (15.3)	11 (7.9)	0.080
Birth weight, grams	1080 (872.5, 1350)	1350 (990, 1498.8)	<0.001
Small for gestational age	23 (23.**5**)	20 (14.**5**)	0.078
**Nutrition**			<0.001
MOM	69 (70.4)	43 (31.1)	
Formula	14 (14.2)	0 (0)	
MOM+Formula	15 (15.3)	24 (17.3)	
MOM+DHM	0 (0)	71 (51.4)	

Categorical variables are presented as frequencies (percentages). Continuous variables are presented as median (IQR) or mean (SD). Abbreviations: GA gestational age, CS Cesarean section, IUGR Intrauterine growth restriction, IDM Infants of diabetic mothers, MOM Mother’s own milk, DHM donor human milk. In bold are significant *p*-values.

**Table 2:** Clinical outcomes and growth-related parameters.

Variable	Epoch-1	Epoch-2	*p*-Value
	(n = 98)	(n = 138)	
Significant respiratory support	95 (96.9)	109 (78.9)	**<0.001**
Surfactant	57 (58.1)	49 (35.5)	**<0.001**
Phototherapy	72 (73.4)	111 (80.4)	0.206
Neonatal complications:			
NEC	18 (18.3)	14 (10.1)	0.069
SIP	2 (2)	4 (2.8)	1
Feeding intolerance	34 (34.6)	31 (22.4)	**0.038**
BSI	24 (24.4)	23 (16.6)	0.146
PVL	2 (2)	5 (3.6)	0.703
IVH	33 (33.6)	42 (30.4)	0.599
Hypoglycemia	16 (16.3)	34 (24.6)	0.124
RDS	92 (93.8)	104 (75.3)	**<0.001**
BPD	55 (56.1)	57 (41.3)	**0.025**
ROP	6 (6.1)	11 (7.9)	0.588
**Growth-related parameters**			
Initiation of enteral feeding (days)	2.3 (±0.9)	1.5 (±0.6)	**<0.001**
Weight change at day 7 (%)	−4.6 (−9.1, 0.1)	−2.8 (−6.9, 1.5)	0.12
Weight change at day 14 (%)	4.9 (−0.7, 13.1)	9 (3.3, 13.6)	**0.019**
Weight change at day 28 (%)	34.8 (22.9, 43.5)	37.6 (24.6, 45.7)	0.356
Time to reach BW (days)	10.5 (±4.8)	9.5 (±4.5)	0.126
Time to full enteral feeding (days)	26.8 (±19.4)	12.8 (±13.6)	**<0.001**
Duration of TPN (days)	20 (10.5, 30)	9 (6, 14)	**<0.001**
Length of hospitalization (days)	72.8 (±39.3)	58.7 (±35.8)	**<0.001**

Categorical variables are presented as frequencies (percentages). Continuous variables are presented as median (IQR) or mean (standard deviation). Abbreviations: NEC Necrotizing enterocolitis, SIP Spontaneous intestinal perforation, BSI Neonatal bloodstream infections, PVL Periventricular leukomalacia, IVH Intraventricular hemorrhage, RDS Respiratory distress syndrome, BPD Bronchopulmonary dysplasia, ROP Retinopathy of prematurity, BW Birth weight, TPN Total parenteral nutrition. In bold are significant *p*-values.

## Data Availability

The data that support the findings of this study are not publicly available due to their containing information that could compromise the privacy of research participants but are available from the corresponding author [J.H.].

## Supplementary Materials

The following supporting information can be downloaded at: https://www.mdpi.com/article/10.3390/nu18203338/s1, Table S1: Predictors in the variability of neonatal percentage of weight change from BW at day 14; Table S2: Predictors in the variability of Length of hospitalization; Table S3: Predictors in the variability of day of reaching full enteral feeding; Table S4: Logistic regression analysis for predictors of NEC.

## Abbreviations

The following abbreviations are used in this manuscript:

VLBW	Very low birth weight
BPD	Bronchopulmonary dysplasia
BSI	Bloodstream infections
BW	Birth weight
CS	Cesarean section
DHM	Donor human milk
GA	Gestational age
HM	Human milk
ICROP	International classification of ROP
IDM	Infants of diabetic mothers
IQR	Interquartile ranges
IUGR	Intrauterine growth restriction
IVH	Intraventricular hemorrhage
LC-PUFA	Long-chain polyunsaturated fatty acids
MOM	Mother’s own milk
NEC	Necrotizing enterocolitis
PVL	Periventricular leukomalacia
RDS	Respiratory distress syndrome
ROP	Retinopathy of prematurity
SD	Standard deviation
SGA	Small for gestational age
SIP	Spontaneous intestinal perforation
TPN	Total parenteral nutrition
