# Supplementary material for: Growth and Clinical Outcomes Before and After Implementation of a Donor Human Milk Program in Very Preterm and/or Very-Low-Birth-Weight Infants
Source: Nutrients. 2026 Oct 12;18(20):3338. doi: 10.3390/nu18203338 (PMC13708150; doi:10.3390/nu18203338)
Supplement: Supplementary file 1 [file nutrients-18-03338-s001.zip › nutrients-4580754-supplementary.pdf]

Supplemental tables:

Table S1: predictors in the variability of neonatal percentage of weight change from BW at day 14

	% of weight change from BW at day 14	
	B Coefficient (95% CI)	p-value
Epochs	0.116 (-0.390-5.157)	0.092
GA	0.566 (1.729-3.300)	**< 0.001**
BW	-0.379 (-0.016- -0.006)	**< 0.001**
Gender	0.123 (0.096-4.944)	**0.042**
Feeding intolerance	-0.048 (-3.800- 1.627)	0.431
Initiation of enteral feeding, day	0.144 (0.130-3.453)	**0.035**
Data is presented as B coefficients with 95% confidence intervals (CI) for the changes (Delta) in percentages in weight. Abbreviations: GA Gestational Age, BW Birth weight.		

Table S2: predictors in the variability of Length of hospitalization

	Length of hospitalization	
	B Coefficient (95% CI)	p-value
Epochs	0.037 (-3.955 - 9.618)	0.412
GA	-0.521 (-10.803 - -6.462)	**< 0.001**
BW	-0.211 (-0.037 - -0.009)	**< 0.001**
Gender	0.047 (-2.364 - 9.527)	0.236
Feeding intolerance	0.020 (-5.150 - 8.538)	0.626
Initiation of enteral feeding, day	0.036 (-2.465 - 5.845)	0.424
NEC	0.114 (3.633 - 21.607)	**0.006**
RDS	-0.051 (-14.465 - 4.219)	0.281
BPD	0.147 (2.927 - 19.335)	**0.008**
Data is presented as B coefficients with 95% confidence intervals (CI) for the changes (Delta) in percentages in weight. Abbreviations: GA Gestational Age, BW Birth weight, NEC Necrotizing enterocolitis, RDS Respiratory distress syndrome, BPD bronchopulmonary dysplasia.		

<u>Table S3</u>: predictors in the variability of day of reaching full enteral feeding

	Day of reaching full enteral feeding	
	B Coefficient (95% CI)	p-value
Epochs	-0.255 (-13.424 - -4.802)	**< 0.001**
GA	-0.150 (-2.387 - 0.057)	0.062
BW	-0.148 (-0.016 - 0.001)	0.072
Gender	-0.034 (-4.980 - 2.553)	0.526
Feeding intolerance	0.155 (1.774 - 10.497)	**0.006**
Initiation of enteral feeding, day	0.024 (-2.063 - 3.101)	0.692
NEC	0.230 (6.195- 17.809)	**< 0.001**
Data is presented as B coefficients with 95% confidence intervals (CI) for the changes (Delta) in percentages in weight. Abbreviations: GA Gestational Age, BW Birth weight, NEC Necrotizing enterocolitis.		

<u>Table S4:</u> Logistic regression analysis for predictors of NEC

	NEC	
	Odds Ratio (95% CI)	p-value
Epochs	1.210 (0.368-3.976)	0.753
GA	1.390 (1.019-1.897)	**0.038**
Gender	0.675 (0.243 -1.877)	0.452
SGA	0.665 (0.182 - 2.426)	0.537
Initiation of enteral feeding, day	1.076 (0.580 -1.994)	0.817
Duration of TPN, day	1.123 (1.073-1.174)	**< 0.001**
Feeding intolerance	3.993 (1.401-11.375)	**0.010**
Data is presented as odds ratios (OR) with 95% confidence intervals (CI) for the morbidities above. Abbreviations: GA Gestational Age, BW Birth weight, TPN total parenteral nutrition.		
